# Aberrant methylation of ERBB pathway genes in sporadic colorectal cancer

**DOI:** 10.1007/s13353-014-0253-6

**Published:** 2014-11-01

**Authors:** Elzbieta Szmida, Pawel Karpiński, Przemyslaw Leszczynski, Tomasz Sedziak, Wojciech Kielan, Paweł Ostasiewicz, Maria M. Sasiadek

**Affiliations:** 1 Department of Genetics, Wroclaw Medical University, ul. Marcinkowskiego 1, 50-368 Wroclaw, Poland; 2 First Department of Surgical Oncology, Lower Silesian Oncology Center, Wroclaw, Poland; 3 Second Department of General and Oncological Surgery, Wroclaw Medical University, Wroclaw, Poland; 4 Department of Pathology, Wroclaw Medical University, Wroclaw, Poland

**Keywords:** Colorectal, Methylation, Illumina 27k, ERBB, PKCB

## Abstract

The ErbB signalling network plays a crucial role in the growth and progression of several cancers, including colorectal cancer (CRC), and includes potentially drug-targetable genes. Oncogenic activation of the ErbB pathway by mutations and focal amplifications have emerged recently as an important predictive marker of the prognosis of CRC patients. However, in contrast to genetic events, little is known about epigenetic alternations of ErbB-associated genes and their impact on gene expression. Genome-wide methylation in sporadic CRCs (*n* = 12) paired with adjacent normal tissues have been previously analysed by Illumina Infinium HumanMethylation27 (HM27) at 27,578 CpG sites. For confirmation of our initial genome-wide analysis, we used a published HM27 dataset (GSE25062). Subsequently, CpG island methylation of selected ErbB pathway-associated genes was assessed on 233 CRC samples using methylation-sensitive polymerase chain reaction (MS-PCR) and analysed along with various genetic factors associated with CRC [epigenotype, *BRAF* and *KRAS* mutations, microsatellite instability (MSI)]. Methylation and expression integration was performed using published datasets including 25 pairs of CRC and normal colon tissues (GSE25062 and GSE25070), and confirmed with real-time PCR. Our previous microarray-based genome-wide DNA methylation analysis of 12 CRCs revealed that four ErbB-associated genes (*PIK3CD*, *PKCΒ*, *ERBB4*, ) were differentially methylated in CRCs. This was further confirmed by statistical re-analysis of an HM27 dataset (GSE25062). Frequent methylation at these loci in tumours was subsequently confirmed by MS-PCR (63 %, 43 %, 43 % and 92 %, respectively). Hypermethylation of *PKCΒ* associated with *KRAS* mutation (*p* = 0.04), whereas hypermethylation of *ERBB4* associated with high-methylation epigenotypes (HME), *BRAF* mutation and MSI (*p* = 0.001, 0.002 and 0.0002, respectively). One of the four analysed genes (*PKCΒ*) was significantly downregulated in CRC tissue, as revealed by real-time PCR and re-analysis of the GSE25062 and GSE25070 datasets. After careful re-analysis of published methylation and expression data, we conclude that methylation of *ERBB4*, *PAK7* and *PIK3CD* has no functional role in CRC carcinogenesis. In contrast, methylation seems to have a potential impact on the biology of colorectal tumours by negatively modulating the expression of *PKCΒ*. Importantly, the relationship between DNA methylation of *PKCΒ* and gene expression may warrant further attention in the context of colon cancer chemoprevention and anti-cancer therapy.

**Electronic supplementary material:**

The online version of this article (doi:10.1007/s13353-014-0253-6) contains supplementary material, which is available to authorized users.

## Introduction

The ErbB signalling network participates in cancer development by the transduction of mitogenic signals (Yarden and Sliwkowski 2001). It plays a crucial role in many pivotal processes, like cell division, migration, adhesion, differentiation and apoptosis. The contribution of the ErbB pathway to such a wide range of roles is possible because its key proteins, receptor tyrosine kinases [*ErbB1* (*HER1*, *EGFR*), *ErbB2* (*HER2*), *ErbB3* (*HER3*), *ErbB4* (*HER4*)], are transmembrane proteins which transfer signals from the cell membranes to other pathways, including Ras/Raf/mitogen-activated protein kinase (MEK) or the phosphatidylinositol 3-kinase (PI3K)/Akt pathway (Lemmon and Schlessinger 2010). Their significant role in the development of neoplasia makes the ErbB protein kinases attractive targets for pharmacological intervention. Several ErbB-targeted inhibitors are currently in use, including trastuzumab (Herceptin) for metastatic breast cancer, cetuximab for metastatic colorectal cancer (CRC) and head and neck cancer, and panitumumab for metastatic CRC (Zhang et al. 2007).

Genetic alterations of the ErbB genes have been commonly observed in various cancers (Wu et al. 2009). The amplification of ErbB tyrosine kinases have been detected in several cancer types, including node-negative breast cancer and bladder cancer. whereas point mutations were described in lung, breast and colon cancer (Andrulis et al. 1998; Engelman et al. 2007; Hoque et al. 2010; Sauter et al. 1993).

Besides genetic alternation, epigenetic changes of the ErbB signalling network and related genes have been reported. These changes include methylation of promoter-associated CpG islands, leading to significant silencing of gene expression (Razin and Cedar 1991). Several studies reported on the methylation of ErbB signalling pathway genes in various cancers, including breast cancer, head and neck cancer, and lung cancer (Das et al. 2010; Scartozzi et al. 2011). However, the overwhelming majority of such studies have dealt with only the *EGFR* gene (Montero et al. 2006; Petrangeli et al. 1995; Scartozzi et al. 2011). Therefore, in this report, we surveyed 233 CRCs for the methylation of four ErbB signalling members (*PIK3CD*, *PKCΒ*, *ERBB4*, *PAK7*) that were initially indicated by our genome-wide DNA methylation analysis and related it to published genome-wide methylation and expression datasets (GSE25062 and GSE25070) (Hinoue et al. 2012; Laczmanska et al. 2013).

## Materials and methods

### Samples

Examination was carried out on 233 samples of primary, sporadic CRCs obtained from the Second Department of General and Oncological Surgery, Wroclaw Medical University, and from the First Department of Surgical Oncology, Lower Silesian Oncology Center, both in Wroclaw. The mean age of CRC patients was 64.9 years (range 35–88 years). The group consisted of 126 males and 107 females. Expression was examined on six pairs of normal and CRC samples.

### Datasets used in this study

Data on methylation profiling (Illumina Infinium 27k) of 12 CRC samples have been published by our group previously (Laczmanska et al. 2013).

The GSE25062 and GSE25070 datasets are part of the same experiment published by Hinoue et al. (2012). GSE25062 consists of genome-wide methylation data (Illumina Infinium 27k) for 125 colorectal tumours and 29 adjacent normal tissues. A minor part of these samples (25 colorectal tumours and matched normal adjacent colonic tissue) have also been analysed for their genome-wide expression profile (HumanRef-8 v3.0, Illumina) and the resulting data were published under accession number GSE25070.

Statistical re-analyses of the methylation and expression data were performed on samples with both types of data available.

### Methods

The selection of four ErbB signalling members (*PIK3CD*, *PKCΒ*, *ERBB4*, *PAK7*) was based on our previously published data (Laczmanska et al. 2013). Briefly, genome-wide methylation at 27,578 CpG sites (spanning 14,495 genes) was examined in 12 CRC samples paired with adjacent, normal colon tissue using the Illumina Infinium HumanMethylation27 (HM27) assay. For additional evaluation of our initial array results, we utilised a large dataset (GSE25062) published by Hinoue et al. (2012).

DNA from tissue was isolated using the Gentra Puregene Tissue Kit (Qiagen, Hilden, Germany), according to the manufacturer’s manual. Bisulphite treatment of 1 μg of genomic DNA obtained from resected frozen tissues was carried out using the EpiTect Bisulfite Kit (Qiagen).

All CRC cases have been previously characterised for various molecular classifiers, including epigenotype, microsatellite instability (MSI), *BRAF* ^V600E^ and *KRAS* codon 12 mutations. Briefly, epigenotyping was performed by the use of seven markers and combined bisulphite restriction analysis (COBRA), as described by Yagi et al. (2010). *BRAF* ^V600E^ mutation in tumour tissues was assessed using the mutant allele-specific polymerase chain reaction (PCR) amplification described by Sapio et al. (2006). Mutations at codon 12 of the *KRAS* gene were detected by PCR–restriction fragment length polymorphism (RFLP), as described by Miranda et al. (2006). Microsatellite instability was determined by pentaplex PCR, using the quasimonomorphic markers as described by Buhard et al. (2006).

Oligonucleotide sequences were designed with the MethPrimer online tool (http://www.urogene.org/cgi-bin/methprimer/methprimer.cgi). The primer sequences and amplification conditions used in this study are described in Table 1. Briefly, PCR was carried out in a 15-μl solution containing 50 ng of the bisulphite-treated DNA, 1× PCR buffer (GeneSys, Wroclaw, Poland), 1.5 mM MgCl_2_, 0.8 mM dNTPs, 0.6 mM forward and reverse primers, and 0.15 U HotStarTaq DNA Polymerase (GeneSys, Wroclaw, Poland). PCR reactions were hot-started at 95 °C for 5 min, subsequently denatured for 30 s at 95 °C, with annealing for 30 s at the appropriate temperature for each primer (Table 1) and an extension for 30 s at 70 °C. Thirty-five cycles were used to amplify the PCR products to the expected sizes in an MJ Mini thermal cycler (Bio-Rad Laboratories, Inc., Hercules, CA, USA). The products were evaluated using 2.5 % agarose gel.

**Table 1:** Primer sequences and annealing temperatures used in this study

Gene	Annealing (°C)	Primer sequences	Product size (bp)
*PAK7*	60	FM 5′GATATTTTTTGGTATAAAAATGCGT3′ RM 5′CAATTACTAAATAAACTACCCCGAT3′	200
		FUM 5′GATATTTTTTGGTATAAAAATGTGT3′ RUM 5′CAATTACTAAATAAACTACCCCAAT3′	200
*PIK3CD*	57,6	FM 5′GAATAATTTTTTTAAAATATGGCGA3′ RM 5′ACAACGAAATAAAAATCCTAAACG3′	257
		FUM 5′GAATAATTTTTTTAAAATATGGTGA3′ RUM 5′CAACAAAATAAAAATCCTAAACACC3′	256
*PKCΒ*	50	FM 5′TCGTTTCGTAGGTTTTTTTATTTTC3′ RM 5′TACCAACTACTTTACATATCGACGC3′	206
		FUM 5′TGTTTTGTAGGTTTTTTTATTTTTGT3′ RUM 5′ACCAACTACTTTACATATCAACACC3′	204
*ERBB4*	60	FM 5′GGTTGTTTTATTTTTATCGTTTTTC3′ RM 5′ACACTTATCCGACGACTACGAT3′	145
		FUM 5′TGTTTTATTTTTATTGTTTTTTGT3′ RUM 5′TCCTCACACTTATCCAACAACTACA3′	147

FM/RM: forward and reverse primers specific to methylated sequence

FUM/RUM: forward and reverse primers specific to unmethylated sequence

Total RNA from the frozen tissues was isolated using the TriPure reagent (Roche Diagnostics, Mannheim, Germany). Transcription RNA to ssDNA was carried out with the Transcriptor First Strand cDNA Synthesis Kit (Roche Diagnostics, Mannheim, Germany), according to the manufacturer’s protocol. Quantitative real-time PCR (QPCR) was conducted using LightCycler 480 Probes Master (Roche Diagnostics, Mannheim, Germany) in a total volume of 10 μl using a LightCycler 480 Real-Time PCR System (Roche Diagnostics, Mannheim, Germany). The PCR conditions were as follows: denaturation at 95 °C for 5 min, followed by a further 50 cycles of denaturation at 95 °C for 10 s, annealing at 58 °C for 30 s and extension 72 °C for 10 s. The sequences of the primer was taken from the Universal ProbeLibrary Assay Design Center for Human (http://www.roche-applied-science.com) and they are: qRT-PKCΒ 5′AGGGATTCCAGTGCCAAGT3′, 5′GAGGCTGGACCCTTGTCAG3′, qRT-ACTB 5′ATTGGCAATGAGCGGTCC3′ and 5′CGTGGATGCCACAGGACT3′. *ACTB* was used as the reference gene. The relative levels of gene expression were performed using the LightCycler 480 Instrument II software with advanced relative quantification for all samples. All experiments were repeated in duplicate.

### Statistical analysis

Linear regression was used to assess differential methylation from HM27 data using MethLAB software (Kilaru et al. 2012). All *p*-values were corrected using Benjamini–Hochberg (B-H) false discovery rate correction. All probes with difference in β-values ≥0.20 and significant B-H-corrected *p*-values between cancer and normal tissues were retained.

The Pearson Chi-squared test (if all expected cell frequencies were ≥5) or Fisher’s exact test was used to test whether the presence of a clinical/molecular correlate is associated with the methylation of a CpG island. The tests used were two-sided. The VassarStats online package was used to carry out the necessary statistical tests and calculate the confidence intervals for the odds ratio (http://vassarstats.net/).

Pearson correlation was used to investigate the correlation between the DNA methylation and expression (Fellows 2012).

Differential expressions were determined by the Cyber-T algorithm (Blazejczyk et al. 2007). All *p*-values were corrected using B-H false discovery rate correction. All probes with significant B-H-corrected *p*-values were retained.

## Results

**Fig. 1:**
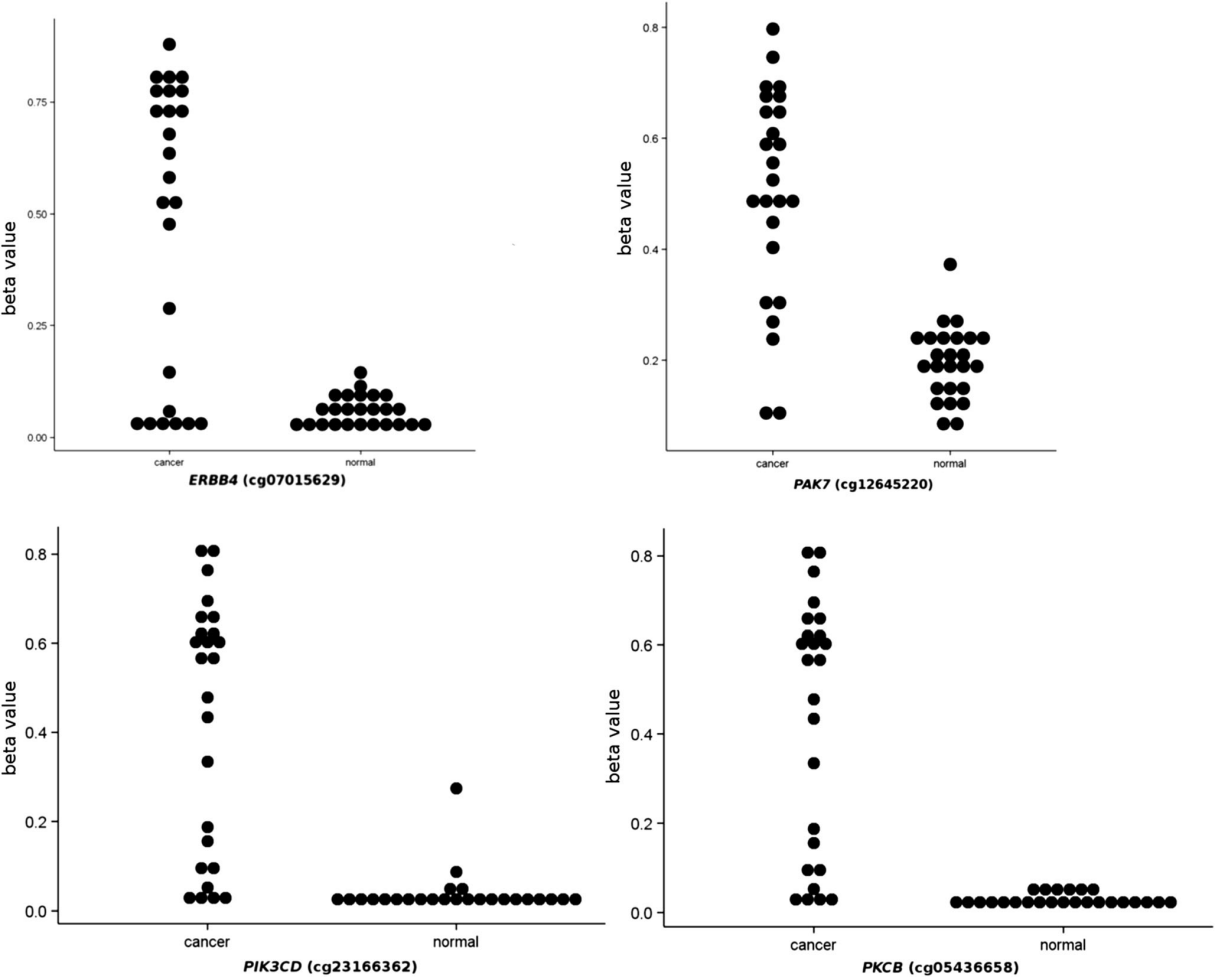
Dot plots of methylation (cancer and normal tissues) measured for *PIK3CD* (cg23166362), *PKCΒ* (cg05436658), *ERBB4* (cg07015629) and *PAK7* (cg12645220). The plots were generated using data from 25 colorectal cancers (CRCs) and paired adjacent normal tissues (GSE25070) (Hinoue et al. 2012)

We have previously described genome-wide methylation at 27,578 CpG sites (spanning 14,495 genes) in 12 CRC samples assessed by the HM27 assay (Laczmanska et al. 2013). This analysis revealed that four ErbB-associated genes (*PIK3CD*, *PKCΒ*, *ERBB4*, *PAK7*) were differentially methylated, as compared to normal control tissues. This was subsequently confirmed by re-analysing a GSE25062 dataset of 25 CRC and 25 adjacent normal tissues (Fig. 1). Subsequently, four genes [*PIK3CD* (cg23166362), *PKCΒ* (cg05436658), *ERBB4* (cg07015629), *PAK7* (cg12645220)] have been selected for further investigation on the group of 233 CRCs that have been previously characterised for various molecular classifiers, including epigenotype, MSI, *BRAF* ^V600E^ and *KRAS* codon 12 mutations (see Supplementary Fig. S1) (Karpinski et al. 2012). Given the uncertainty of intermediate- and low-methylation epigenotypes reported previously by our group, we decided to combine both epigenotypes into one group (IME/LME) (Karpinski et al. 2012).

An overview of the methylation frequencies in the studied CRC samples is given in Table 2. Overall, the incidence of hypermethylation at the *PAK7* genes was the highest (91 %), whereas hypermethylation at the *ERBB4* and *PKCΒ* genes was the lowest (43 % each). With regard to the clinicopathological characteristics (sex, tumour localisation), no association could be observed. However, there was a relationship between the *PKCΒ* and *KRAS* mutations (*p* = 0.042) and a significant association between *ERBB4* methylation and *BRAF* ^V600E^ mutation, MSI (*p* = 0.001 and *p* = 0.002, respectively) and high-methylation epigenotypes (HME) (*p* = 0.0002).

**Table 2:** Methylation of selected ErbB signalling genes, and clinical and molecular features among 233 colorectal cancers (CRCs)

	*PIK3CD*		*p*-Value	*PKCΒ*		*p*-Value	*ERBB4*		*p*-Value	*PAK7*		*p*-Value
	+	−		+	−		+	−		+	−	
Male	78	48		55	71		59	67		113	13	
Female	68	39	0.79	46	61	0.92	42	65	0.24	101	6	0.19
Distal	99	67		73	93		68	98		155	11	
Proximal	47	20	0.13	28	39	0.76	33	34	0.24	59	8	0.17
MSI	14	5		10	9		15	4		18	1	
MSS	132	82	0.17	91	123	0.39	86	128	**0.001**	196	18	1
*BRAF*+	11	9		9	11		15	5		18	2	
*BRAF*−	135	78	0.45	92	121	0.88	86	127	**0.002**	196	17	1
*KRAS*+	44	23		36	31		30	37		62	5	
*KRAS*−	102	64	0.54	65	101	**0.042**	71	95	0.77	152	14	0.8
HME	17	9		14	12		20	6		24	2	
IME/LME	127	78	0.72	87	120	0.25	81	126	**0.0002**	190	17	1
All CRCs (%)	146/233 (63)	87		101/233 (43)	132		101/233 (43)	132		214/233 (91)	19	

**Fig. 2:**
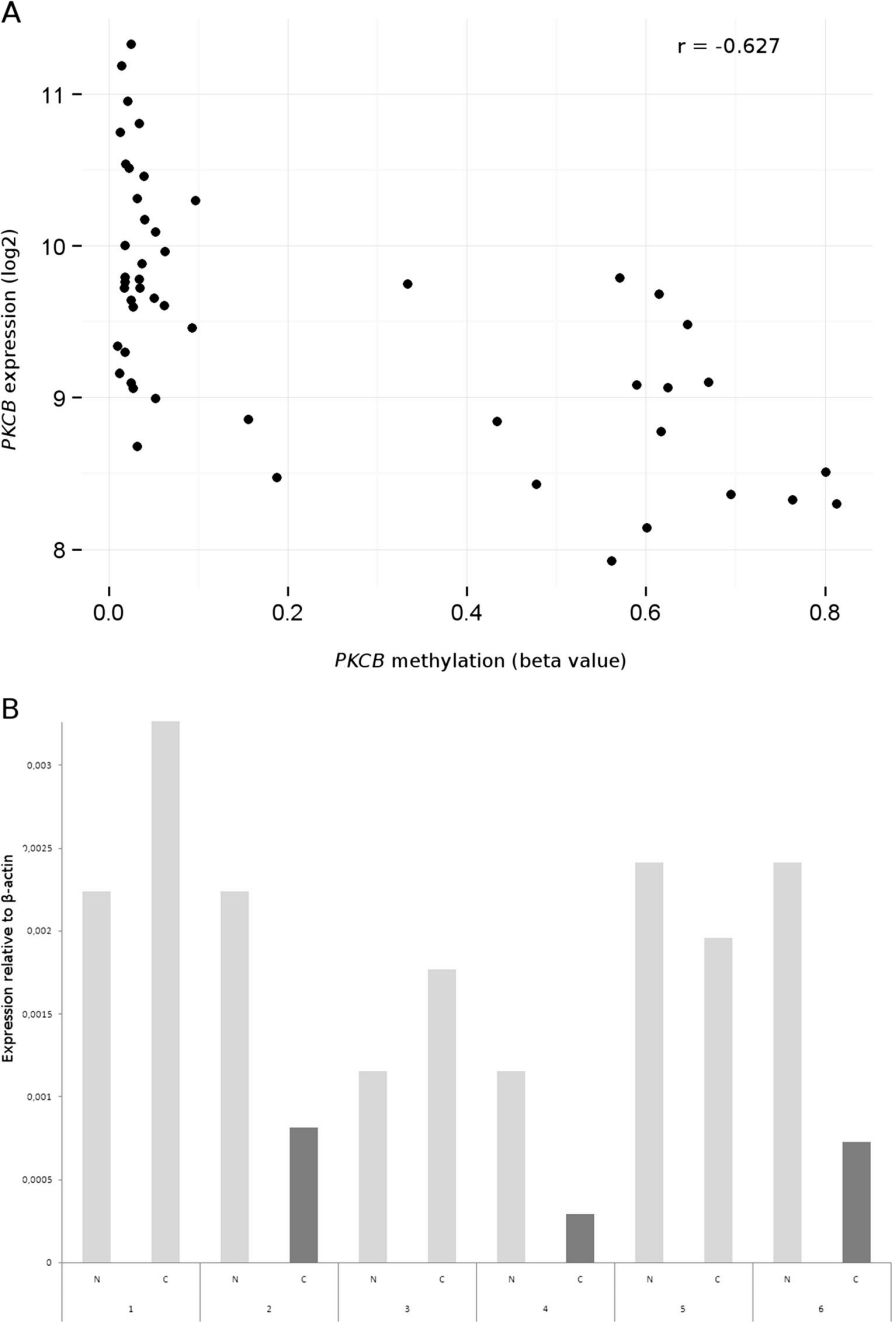
**a** Correlation plot between DNA methylation and gene expression of the *PKCΒ* gene. The plot was generated using data from 25 CRCs and paired adjacent normal tissues (GSE25070 and GSE25070) (Hinoue et al. 2012). **b** Relative expression of the *PKCΒ* gene in six selected normal (N) and tumour (C) pairs. The *dark grey bars* indicate tumours with *PKCΒ* methylation. A decrease in the relative expression level could be noticed in tumours with *PKCΒ* methylation

To explore the links between differential methylation and the expression of selected genes, we utilised data obtained by Hinoue et al. with the Illumina HumanRef-8 v3.0 Expression BeadChip that contain the transcripts level for 25 CRCs and paired adjacent normal tissues (GSE25070) (Hinoue et al. 2012). In this dataset, significant downregulation of expression between cancerous and normal tissue was revealed for one of four genes (*PKCΒ*). Subsequently, Pearson correlation analysis of methylation (GSE25062) and expression (GSE25070) data in 25 paired tissues has shown a negative correlation between methylation and expression for the *PKCΒ* gene (*r* = −0.627) (Fig. 2a). We confirmed the results of re-analysis by performing real-time PCR on six selected normal (N) and tumour (C) pairs (Fig. 2b).

## Discussion

In this study, we assessed the methylation of four members of the ErbB signalling network (*PIK3CD*, *PKCΒ*, *ERBB4*, *PAK7*) and related it to a number of important clinical and molecular features in order to assess whether methylation of these genes is dependent on defined molecular/clinical features that may promote increased methylation of a given gene. Several studies have shown alternations in the expression of the candidate genes in various cancers, including gastrointestinal and other epithelial cancers, whereas few, if any, focused on epigenetic changes of the above-mentioned genes in CRC (Sawyer et al. 2003).

*ERBB4* (*HER4* receptor tyrosine kinase) methylation has been previously reported in breast carcinomas and significantly associated with worse patient prognosis (Das et al. 2010). In our study, we identified 43 % CRC samples with *ERBB4* promoter methylation which correlated with HME tumour status, MSI and *BRAF* ^V600E^ mutation. Given that MSI and *BRAF* ^V600E^ mutation are molecular correlates specific for HME tumours, it can be postulated that *ERBB4* promoter methylation is, rather, related to HME than specifically to the two other above-mentioned molecular correlates (Yagi et al. 2010). Concerning the biological role of *ERBB4* methylation, recent studies demonstrated that the *ERBB4* locus is occupied by the H3K27me3 (histone 3 lysine 27 trimethylation) histone mark in normal colon tissue (Enroth et al. 2011). Given that the H3K27me3-associated genes tend to be transcriptionally silent in normal tissue and hypermethylated in tumours, it can be speculated that *ERBB4* methylation has no biological meaning in colorectal carcinogenesis (Kouzarides 2007).

*PAK7* (p21-activated kinase 7, also known as *PAK5*) belongs to a family of six genes that activate the cell-survival signalling pathway. Unlike *PAK4*, the deletion of *PAK7* is not sufficient to induce tumours in mice; however, *PAK7* driver mutations have been described in cancers (Furnari et al. 2013). Two recent studies demonstrated that: (i) *PAK7* is overexpressed during colorectal and gastric cancer progression (Gong et al. 2009; Gu et al. 2013); (ii) *PAK7* knock-down suppresses gastric cell lines proliferation (Gu et al. 2013). In contrast, we demonstrated very frequent methylation of *PAK7* in our cases (91 %). This finding was also supported by our re-analysis of the published HM27 dataset (GSE25062) and data obtained in CRCs by genome-wide methylation analysis in three other studies where *PAK7* has been found to be frequently methylated (Sproul et al. 2012; Xu et al. 2012). This suggests that the *PAK7* mRNA levels may not be determined by methylation extent alone or the location of biologically relevant methylation has not been properly addressed; thus, further studies are needed in order to elucidate the relation of methylation and *PAK7* expression in CRC (van Vlodrop et al. 2011).

*PIK3CD* encodes the delta isoform of phosphoinositide-3 kinase (p110δ) that transmits signals inside cells by phosphorylating inositol lipids in cellular membranes (Tzenaki and Papakonstanti 2013). *PIK3CD* is expressed mainly in leucocytes; however, the expression of *PIK3CD* was also evidenced in non-leucocyte cancer cell lines, including breast carcinoma, melanoma and glioma (Kok et al. 2009; Sawyer et al. 2003). Notably, in the same study, the expression of *PIK3CD* in two colorectal cell lines was not evidenced (Sawyer et al. 2003). This was also confirmed in our re-analysis of the published expression dataset (GSE25070). As in the case of *ERBB4* mentioned above, a recent study revealed occupation of the *PIK3CD* locus by H3K27me3 histone mark in normal colon tissue, which likely results in *PIK3CD* being prone to hypermethylation in tumours without a direct impact on colorectal carcinogenesis (Enroth et al. 2011).

*PKC* beta (*PKCΒ*) is one of the protein kinase C isoforms involved in regulating cell proliferation and survival. *PKCΒ* is a component of the VEGF signalling pathway, which may promote tumour-directed angiogenesis (Spalding et al. 2008). Upregulated *PKCΒ* has been found in multiple human tumours, including CRCs (Gökmen-Polar et al. 2001). It has been shown that enhanced expression of *PKCΒ* induces tumorigenesis in mice colon (Graff et al. 2005). Recently, it has been found that *PKCΒ* hypermethylation is dependent on transcription factor *PROX1* (prospero-related homeobox 1) high expression levels in colon cancer (Hagiwara et al. 2012). In a recent study, Skog et al. (2011) found that ~30 % of CRCs displayed high *PROX1* expression, which was associated with poor patient outcome. In our study, we found a comparable frequency of *PKCΒ* methylation (43 % of cases). Given the inter-relation between *PROX1* expression and *PKCΒ* methylation, together with the importance of *PROX1* expression as the CRC prognostic factor, *PKCΒ* methylation may be potentially utilised as the indirect *PROX1* expression marker in CRC.

We demonstrated that *PKCΒ* methylation is more frequent in tumours with *KRAS* mutation (Table 2; *p*-value ≤0.04). Interestingly, Calcagno et al. indicated that *KRAS* mutation inhibits expression of *PKCΒ* in mouse distal colon. Thus, it can be speculated that *PKCΒ* methylation observed in our study may be dependent, to some extent, on the *KRAS* mutation status.

Most notably of all, enzastaurin (a *PKCΒ* inhibitor) has shown promising results as an effective and selective suppressor of colon tumour proliferation in a mouse model (Graff et al. 2005). Therefore, *PKCΒ* has been recognised as a potential target for the chemoprevention of CRC (Glimelius et al. 2010). In our study, we have shown the negative effect of methylation on *PKCΒ* expression. In this light, enzastaurin-dependent chemoprevention may have little or no effect in patients with decreased *PKCΒ* expression. It might be of particular interest to further evaluate the methylation of *PKCΒ* as a potential marker of enzastaurin chemoprevention.

## Conclusions

In this study, we demonstrated frequent methylation of the ErbB signalling network (*PIK3CD*, *PKCΒ*, *ERBB4*, *PAK7*) in colorectal cancer (CRC). After careful re-analysis of published methylation and expression data, we conclude that the methylation of *ERBB4*, *PAK7* and *PIK3CD* has no functional role in CRC carcinogenesis. In contrast, methylation seems to have a potential impact on the biology of colorectal tumours by negatively modulating the expression of *PKCΒ*. Given the role of *PKCΒ* as the potential target for anti-cancer therapy, further investigation of *PKCΒ* methylation and expression in CRC could be of great importance for the development of future therapeutic strategies.

## Electronic supplementary material

Below is the link to the electronic supplementary material.

Supplementary Fig. S1 MSP analysis of selected genes in CRC tissues. “m” denotes methylation-specific reaction, whereas “um” indicates reaction with primers specific for unmethylated alleles. *1*–*5* colorectal cancer samples; *6* fully methylated Jurkat DNA (New England Biolabs); *7* whole-genome amplified human DNA (methylation-negative control); *8* ddH_2_O (GIF 1,819 kb)

High-resolution image (TIFF 2,300 kb)
