# Supplementary figures and images for: Aberrant methylation of ERBB pathway genes in sporadic colorectal cancer
Source: J Appl Genet. 2014 Nov 1;56(2):185–92. doi: 10.1007/s13353-014-0253-6 (PMC4412553; doi:10.1007/s13353-014-0253-6)

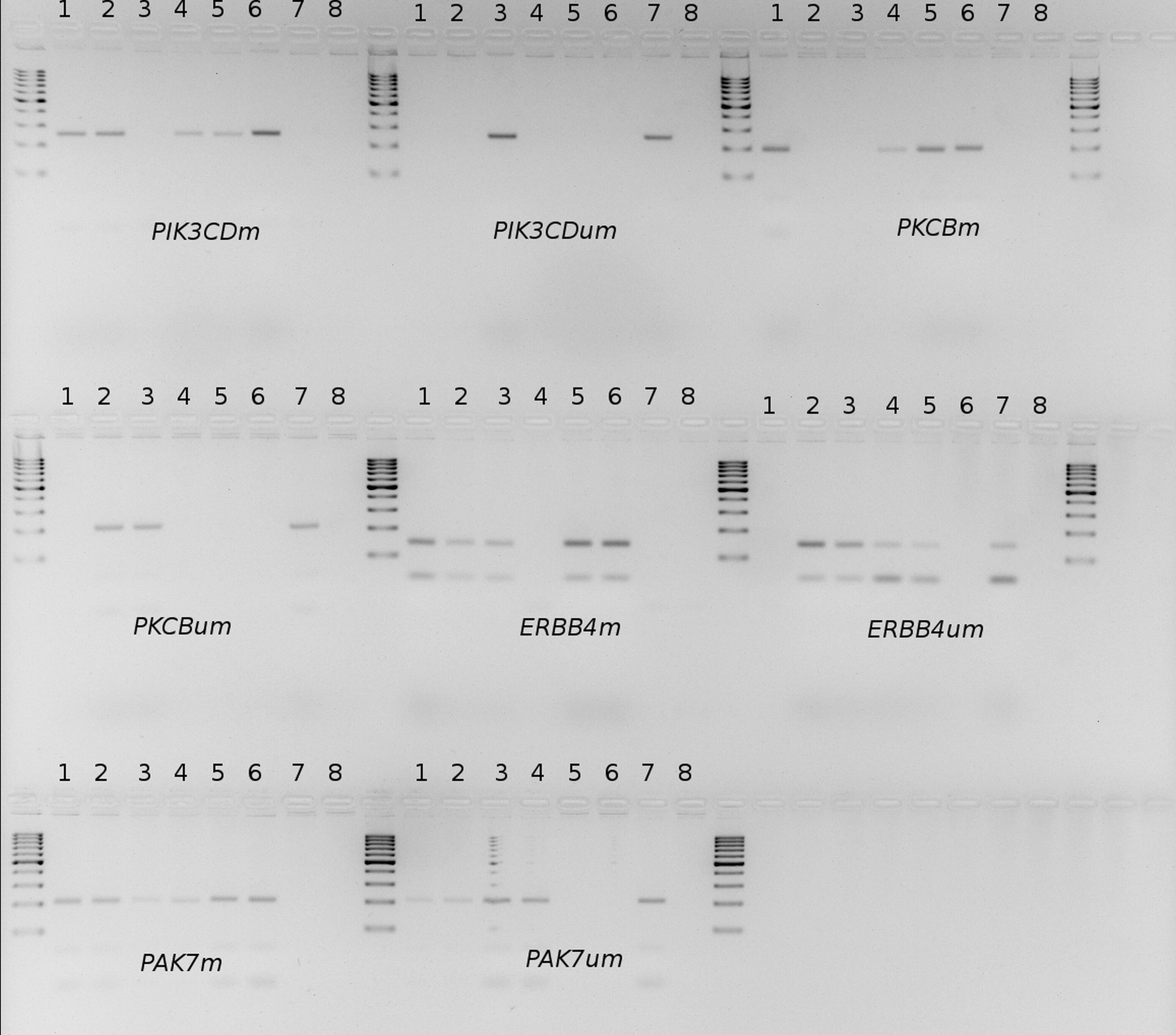

Supplement: Supplementary file 1 — MSP analysis of selected genes in CRC tissues. “m” denotes methylation-specific reaction, whereas “um” indicates reaction with primers specific for unmethylated alleles. 1–5 colorectal cancer samples; 6 fully methylated Jurkat DNA (New England Biolabs); 7 whole-genome amplified human DNA (methylation-negative control); 8 ddH2O (GIF 1,819 kb) [file 13353_2014_253_Fig3_ESM.gif]
